# Depression and quality of life among Afghan healthcare workers: A cross-sectional survey study

**DOI:** 10.1186/s40359-023-01059-9

**Published:** 2023-01-30

**Authors:** Abdul Qadim Mohammadi, Ahmad Neyazi, Vanya Rangelova, Bijaya Kumar Padhi, Goodness Ogeyi Odey, Molly Unoh Ogbodum, Mark D. Griffiths

**Affiliations:** 1 Mental Health Ward, Herat Regional Hospital, Herat, Afghanistan; 2 Afghanistan Center for Epidemiological Studies, Herat, Afghanistan; 3 Department of Epidemiology and Disaster Medicine, Faculty of Public Health, Medical University Plovdiv, Plovdiv, Bulgaria; 4 Department of Community Medicine and School of Public Health, Postgraduate Institute of Medical Education and Research (PGIMER), Chandigarh, India; 5 Department of Public Health, College of Medicine, University of Calabar, Calabar, Nigeria; 6 Department of Psychology, Nottingham Trent University, Nottingham, UK

**Keywords:** Depression, Quality of life, Healthcare workers, Afghanistan

## Abstract

**Background:**

According to the World Health Organization, approximately 280 million individuals worldwide suffer from depression. One occupational group that is more prone to mental health issues is healthcare workers (HCWs). However, very little is known about the mental health of HCWs in Afghanistan. Therefore, the present study examined depression, quality of life (QOL), and related factors among Afghan HCWs.

**Methods:**

A cross-sectional survey was administered in June 2022 among healthcare workers (N = 299) in the Herat province of Afghanistan. The survey examined depression, its risk factors and predictors among HCWs.

**Results:**

Of the 299 participants, 73.6% of them reported depression symptoms. Low monthly income, working in a private hospital, and being a cigarette smoker were some of the main variables associated with depression symptoms among Afghan HCWs. Multiple regression analysis indicated that field of work (aOR = 3.774, *p* = 0.0048), monthly income (aOR = 0.746, *p* = 0.0088), job type (aOR = 8.970, *p* < 0.0001), cigarette smoking (aOR = 2.955, *p* = 0.0069), a bad event happening during the past month (aOR = 2.433, *p* = 0.0157), physical domain of quality of life (aOR = 0.966, *p* = 0.0186), and psychological domain of quality of life (aOR = 0.950, *p* = 0.0005) were significantly associated with depression symptoms.

**Conclusion:**

The prevalence of depression symptoms is high among healthcare workers in the Herat province of Afghanistan. One of the variables found to have a major impact on the prevalence of depression was their monthly income. Considering its impact on quality of life and the overall quality of healthcare services, the government should implement regular screening for depression, psychological counselling services, and psychiatric treatment for vulnerable healthcare workers.

## Introduction

According to the World Health Organization (WHO), approximately 280 million individuals worldwide suffer from depression [1]. Depression is a common mental health disorder worldwide and causes affected individuals to suffer greatly and perform poorly educationally and/or occupationally [1]. One of the high-risk groups for poor mental health outcomes globally is healthcare workers (HCWs). More specifically, studies have shown that they experience high levels of psychological distress and burnout such as depression, anxiety, and poor sleep quality due to their profession [2, 3]. This is associated with distressing symptoms such as restlessness, anger, frustration, insomnia and fatigue [2]. In high-income countries in 2015, the prevalence of depression among HCWs ranged from 21.53% to 32.77%, which was much higher than the global average of 4.4% [3].

In one study, more than 50% of Egyptian HCWs reported having depression symptoms. The authors speculated that this was most likely due to the country's high workload requirements, chronic staffing shortages, and low HCW wages [3]. Similarly, between 2012 and 2022, 22.0%–45.3% of Iranian HCWs reported having depressive symptoms [3]. Long-term exposure to high pressure has severe effects on HCWs as well as the potential to undermine patient safety and the standard of care they deliver. Patient unhappiness, high HCW turnover rates, medical errors, and related financial expenditures may arise from this [3]. Additionally, stress is widespread among working populations globally, and when different work groups like doctors, nurses, or other members of the health workforce are taken into account, depression prevalence among HCWs varies greatly [4]. Anxiety, fear, frustration, loneliness, fury, boredom, sadness, stress, and maladaptive behaviors like avoidance, emotional discomfort, and defensive reactions are among the psychological reactions of HCWs at the forefront of service delivery [5].

Depression is recognized to have a negative impact on quality of life, productivity, and job performance [4]. The WHO stated that “a person’s view of their place in life in the context of the culture and value systems they live in and in connection to their goals, aspirations, standards, and worries” [5] is how they determine the quality of their life. HCWs must manage stresses at work in addition to stressors in their personal lives, and they are subject to extremely high levels of academic and professional stress [2]. Many obstacles exist in places like Afghanistan (where the current study was conducted) that have a negative impact on mental health. This includes social stigma, healthcare disparities, a lack of funding for mental health services, political unrest and conflict, and HCW shortages [6]. Low resilience negatively impacts the work performance, health condition, and quality of life of HCWs because depression impairs the ability to deal with stressful events [5]. As a result, this directly affects the standard of medical care provided to patients [5].

Medical professionals must react quickly and accurately to the requirements of patients and their families, making their work challenging. In the medical field, shift work, night work, and long work hours are all very common schedules [7]. Numerous studies have shown that prolonged exposure to work-related stress can cause depression [7, 8]. This is especially common in emergency departments where employees are under tremendous psychological strain from their demanding workloads, long shifts, and high-risk environments. The intensity of depression symptoms and low quality of life (QOL) experienced by HCWs are influenced by perceived workplace culture, the accessibility of support, and static and dynamic personal factors [9]. A person's physical health, psychological health, level of independence, social connections, surroundings, and spirituality are all part of their QOL, which is a broad notion. In light of this, low QOL is equally correlated with both depression and mental illness [7].

HCWs often have to respond to demanding and unforeseen medical emergencies, which may be compounded by staff shortages, but their response is unique and multi-factorial. Further investigation of the complex relationships among specific job tasks and responsibilities, work conditions and culture, personal and situational risks, protective factors, and general mental health is critical [9]. It has been found that healthcare workers (including doctors, nurses, and paramedics such as ambulance workers) are prone to depression, anxiety, burnout syndrome, and post-traumatic stress disorder [10]. Many socioeconomic and psychological factors are reported to be associated with the development of stress, anxiety, and depression and these challenges can have repercussions on the already limited healthcare workforce and may exacerbate HCW shortages [2, 10].

The WHO declared that managing HCWs’ mental health and psychosocial wellbeing is as important as managing their physical health in facing workplace stressors [6]. The paucity of high-quality data on mental health problems and the lack of qualified human resources have hampered the development of cost-effective strategies and interventions to address the growing challenge of mental health in Afghanistan [6]. To date, there is a scarcity of data concerning the mental health and quality of life of HCWs in Afghanistan. Therefore, the present study examined depression, QOL, and related factors among Afghan HCWs.

## Methods

### Study design and participants

The present study was a cross-sectional survey conducted among healthcare workers between June 5, 2022, and June 22, 2022, in Herat city (Afghanistan). Print-based surveys were distributed to the participants to complete, and data were collected utilizing a convenience cluster sampling method. More specifically, governmental and private hospitals were selected and HCWs working in those hospitals who agreed to participate in the study completed the survey with each hospital being considered a cluster. Validated psychometric instruments were used included in the survey to assess depression symptoms as well as the quality of life of participants. A total of 500 HCWs in governmental hospitals were approached face-to-face and were asked to take part in the study, and a total of 299 HCWs voluntarily participated in the present study (response rate = 59.8%). Providing informed consent and being healthcare worker at the hospital were the pre-requisites for participation in the study.

### Measures

The survey used in the present study consisted of three sections: socio-economic information, screening for depression, and quality of life assessment. Socio-economic information included questions on age, gender, marital status, number of children, residency, the field of work, economic status, monthly income, job type, number of patients per day, and cigarette smoking.

For the screening of depression, the 19-item Dari version of the Center for Epidemiological Studies–Depression Scale was used [11]. The scale comprises three main groups of items: the negative items group (e.g., *“I felt everything I did was an effort”*), the positive items group (e.g., *“I enjoyed life”*), and the interpersonal relationship items group (e.g., *“I felt people dislike me”*). All the items are scored on a four-point scale from 0 (*“rarely or none of the time/less than one day during the past week”*) to 3 (*Most of all of the time/5–7 days during the past week*) and scores range from 0 to 60. The cut-off scores were: 0–15 was considered as not having depression symptoms, and a score of 16 or over was considered presence of depression symptoms. The Cronbach’s alpha in the present study was 0.87.

To assess the quality of life, the 26-item Dari version of the World Health Organizations Quality of Life-Bref (WHOQOL-Bref 26) assessment was used [12]. The scale assesses the quality of life in four domains: physical health, psychological health, social relationships, and environment. All of the items (e.g., “*To what extent do you feel that physical pain prevents you from doing what you need to do?”*) are scored on a five-point scale from 1 (*not at all*) to 5 (*an extreme amount*). Raw scores were converted into the transformed score to range within 0–100 in order to make it comparable with WHOQOL-100. For each domain of the WHOQOL-Bref 26, a total score of less than 46 was considered a low quality of life. Scores between 46 and 65 were considered a moderate quality of life. Scores higher than 65 were considered a high quality of life [13]. The Cronbach’s alpha in the present study was 0.85.

### Statistical analysis

Microsoft Excel was used to create the database for further analysis. Cross-tabulation was carried out to obtain the numbers and percentages of the socioeconomic variables, depression, and quality of life of participants in each domain. To evaluate the relationship between different variables, chi-square tests were used. Multiple logistic regression analysis was used to compute the adjusted odds ratios (aOR) and their 95% confidence intervals (95% CI). A *p*-value of less than 0.05 was considered significant in the present study. STATA (version 17.0) was used for all statistical analysis.

## Results

**Fig. 1:**
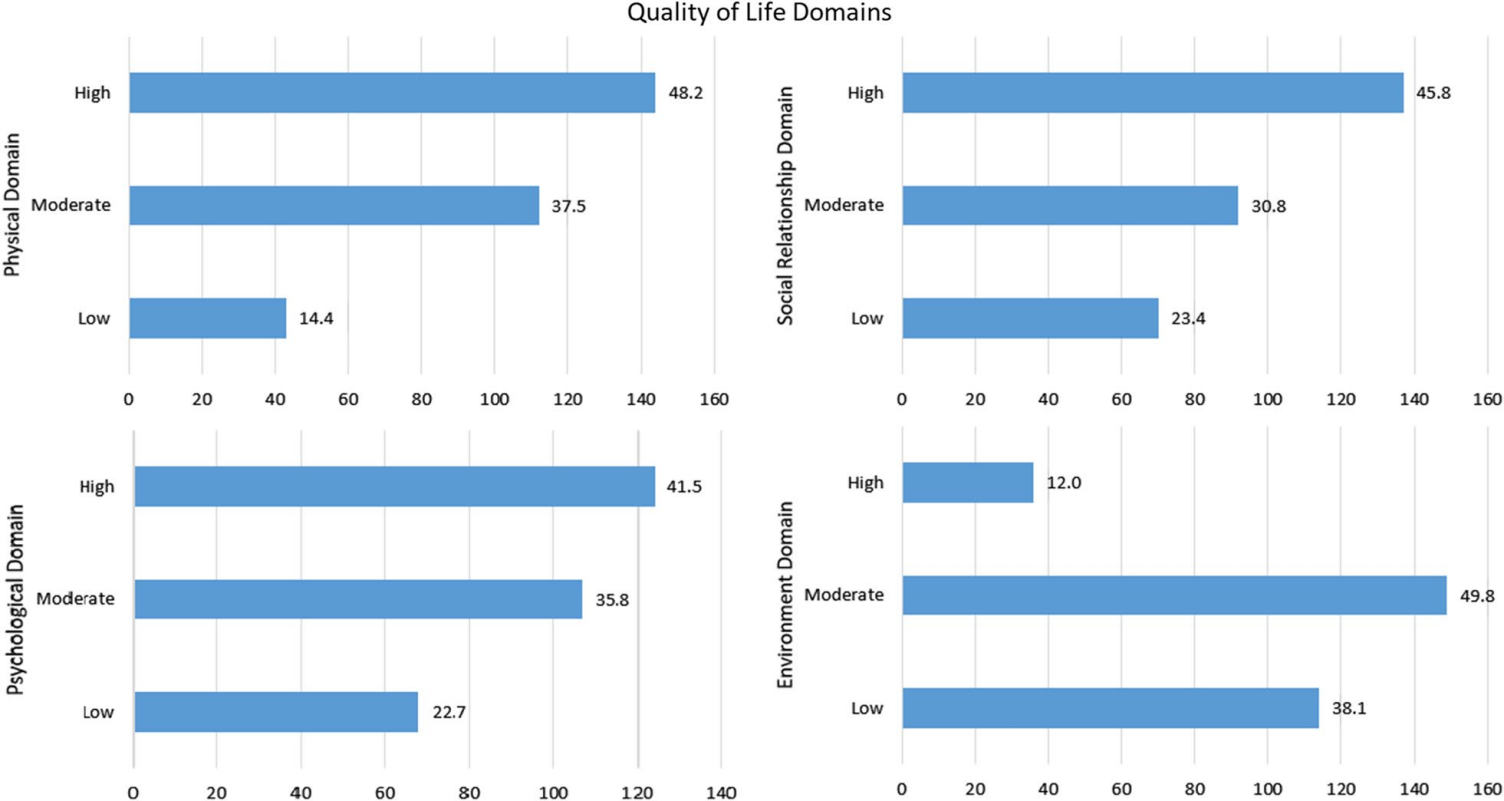
Quality of life of healthcare workers (Herat, Afghanistan-2022)

A total of 299 healthcare workers participated in the present study with an age range of 18–64 years. The mean age of the participants was 30.88 years. More than half of the participants were male (60.5%). Almost three-quarters of the participants were married (74.6%). Over two-thirds of the participants were doctors or residents (69.9%). Almost half of the participants reported that their monthly income was less than the equivalent of $100 (US) or were working as volunteers (48.5%). Two-thirds of the participants were from governmental hospitals (65.6%) (Table 1). The proportion of participants who had a high quality of life in the four domains was as follows: physical domain (48.2%), psychological domain (41.5%), social relationship domain (45.8%), and environment domain (12.0%) category (Fig. 1).

**Table 1:** Characteristics distribution of the study sample (Herat, Afghanistan-2022)

Characteristic	Categories	Number (N)	Percentage (%)
Age group	18–28-years	149	49.8
	29–64-years	150	50.2
Gender	Male	181	60.5
	Female	118	39.5
Marital status	Single	73	24.4
	Married	223	74.6
	Widow	2	0.7
	Divorced	1	0.3
Number of children	None	123	41.1
	1–5 child	162	54.2
	5–12 child	14	4.7
Residency	Urban	271	90.6
	Rural	28	9.4
Field of work	Doctors	209	69.9
	Others	90	30.1
Economic status	High income	102	34.1
	Middle income	166	55.5
	Low income	31	10.4
Monthly income	Less than $100	145	48.5
	$100–$200	35	11.7
	$200–$300	58	19.4
	$300–$400	30	10.0
	$400–$500	9	3.0
	$500–$600	11	3.7
	More than $600	11	3.7
Job type	Private	103	34.4
	Governmental	196	65.6
Number of patients per day	Less than 30	169	56.5
	31–50	54	18.1
	More than 50	76	25.4
Cigarette smoking	Non-smoker	156	52.2
	Ex-smoker	134	44.8
	Current smoker	9	3.0
Event happened during last month	Yes	130	43.5
	No	169	56.5
Total		299	100.0

Three-quarters of the participants were found to have symptoms of depression (73.6). Four-fifths of participants who were single had symptoms of depression (80.8%) and 86.7% of participants who were not dental or medical doctors (nurses, midwives) had symptoms of depression. There was a significant relationship between the field of work, monthly income, job type, and smoking with the presence of depression symptoms (Table 2).

**Table 2:** Association of depression with participants socio-demographic characteristics (Herat, Afghanistan-2022)

Characteristic	Categories	Mental health		*p*-value
		Normal	Depressed	
		N (%)	N (%)	
Age group	18–28-year-old	36 (24.2)	113 (75.8)	0.377
	29–64-year-old	43 (28.7)	107 (71.3)	
Gender	Male	47 (26.0)	134 (74.0)	0.825
	Female	32 (27.1)	86 (72.9)	
Marital status	Single	14 (19.2)	59 (80.8)	0.272
	Married	65 (29.1)	158 (70.9)	
	Widow	0 (0.0)	2 (100.0)	
	Divorced	0 (0.0)	1 (100.0)	
Number of children	None	26 (21.1)	97 (78.9)	0.166
	1–5 child	50 (30.9)	112 (69.1)	
	5–12 child	3 (21.4)	11 (78.6	
Residency	Urban	74 (27.3)	197 (72.7)	0.280
	Rural	5 (17.9)	23 (82.1)	
Field of work	Doctors	67 (32.1)	142 (67.9)	**0.001**
	Others	12 (13.3)	78 (86.7)	
Economic status	High income	32 (31.4)	70 (68.6)	0.056
	Middle income	44 (26.5)	122 (73.5)	
	Low income	3 (9.7)	28 (90.3)	
Monthly income	Less than $100	14 (9.7)	131 (90.3)	** < 0.001**
	$100–$200	8 (22.9)	27 (77.1)	
	$200–$300	22 (37.9)	36 (62.1)	
	$300–$400	17 (56.7)	13 (43.3)	
	$400–$500	5 (55.6)	4 (44.4)	
	$500–$600	7 (63.6)	4 (36.4)	
	More than $600	6 (54.5)	5 (45.5)	
Job type	Private	10 (9.7)	93 (90.3)	** < 0.001**
	Governmental	69 (35.2)	127 (64.8)	
Number of patients	Less than 30	47 (27.8)	122 (72.2)	0.650
	31–50	15 (27.8)	39 (72.2)	
	More than 50	17 (22.4)	59 (77.6)	
Cigarette smoking	Non-smoker	68 (43.6)	88 (56.4)	** < 0.001**
	Ex-smoker	9 (6.7)	125 (93.3)	
	Current smoker	2 (22.2)	7 (77.8)	
Bad event happened during last month	Yes	24 (18.5)	106 (81.5)	**0.001**
	No	55 (32.5)	114 (67.5)	
Total		79 (26.4)	220 (73.6)	

significantly associated values are in bold

Over four-fifths of participants who rated their quality of life as very poor (84.6%) had depression symptoms. Almost all participants with low quality of life in the physical domain (97.7%) had depression symptoms and 94.1% of participants with low quality of life in the psychological domain had depression symptoms (Table 3). Multiple logistic regression was run to predict depression comprising the following variables: field of work, monthly income, job type, cigarette smoking, bad events happening during the past month, and quality of life domains. Analysis indicated that field of work (OR = 3.774, *p* = 0.0048), monthly income (OR = 0.746, *p* = 0.0088), job type (OR = 8.970, *p* < 0.0001), cigarette smoking (OR = 2.955, *p* = 0.0069), a bad event happening during the past month (OR = 2.433, *p* = 0.0157), physical domain of quality of life (OR = 0.966, *p* = 0.0186), and psychological domain of quality of life (OR = 0.950, *p* = 0.0005) were significantly associated with depression symptoms. These variables significantly predicted depression symptoms. All the variables significantly contributed to the regression model except for the social relationship domain and the environment domain related to the quality of life (Table 4).

**Table 3:** Association of quality of life of participants with presence of depression (Herat, Afghanistan-2022)

Quality of life	Categories	Mental health		*p*-value
		Normal	Depressed	
		N (%)	N (%)	
How would you rate your quality of life?	Very poor	2 (15.4)	11 (84.6)	0.002
	Poor	0 (0.0)	11 (100.0)	
	Neither poor nor good	10 (14.1)	61 (85.9)	
	Good	50 (36.2)	88 (63.8)	
	Very good	17 (25.8)	49 (74.2)	
How satisfied are you with your health?	Very dissatisfied	3 (9.4)	29 (90.6)	< 0.001
	Dissatisfied	0 (0.0)	35 (100.0)	
	Neither satisfied nor dissatisfied	11 (10.9)	90 (89.1)	
	Satisfied	43 (49.7)	44 (50.6)	
	Very satisfied	22 (50.0)	22 (50.0)	
Physical domain	Low	1 (2.3)	42 (97.7)	< 0.001
	Moderate	23 (20.5)	89 (79.5)	
	High	55 (38.2)	89 (61.8)	
Psychological domain	Low	4 (5.9)	64 (94.1)	< 0.001
	Moderate	21 (19.6)	86 (80.4)	
	High	54 (43.5)	70 (56.5)	
Social relationship domain	Low	8 (11.4)	62 (88.6)	0.001
	Moderate	23 (25.0)	69 (75.0)	
	High	48 (35.0)	89 (65.0)	
Environment domain	Low	17 (14.9)	97 (85.1)	0.001
	Moderate	48 (32.2)	101 (67.8)	
	High	14 (38.9)	22 (61.1)	
Total		79 (26.4)	220 (73.6)

**Table 4:** Multiple logistic regression analysis of depression on participants’ characteristics and their quality of life (Herat, Afghanistan-2022)

Variable	aOR [95% CI]	*p*-value
*Field of work*		
Doctors	Reference	
Non-doctors	3.774 [1.501, 9.489]	0.0048
*Monthly income*		
Low income	Reference	
High income	0.746 [0.599, 0.929]	0.0088
*Job type*		
Governmental	Reference	
Private	8.970 [3.207, 25.092]	0.0000
*Cigarette smoking*		
Non smoking	Reference	
Smoking	2.955 [1.347, 6.484]	0.0069
*Bad event happened during past month*		
Yes	2.433 [1.183, 5.006]	0.0157
Physical domain	0.966 [0.938, 0.994]	0.0186
Psychological domain	0.950 [0.922, 0.978]	0.0005
Social relationship domain	1.000 [0.979, 1.023]	0.9722
Environment domain	0.998 [0.971, 1.026]	0.8870

## Discussion

To the best of the authors’ knowledge, the present study is the first to examine the prevalence of depression symptoms and its impact on the quality of life of healthcare workers (HCWs) in Afghanistan. Close to three-quarters of the HCWs were found to have mild to severe symptoms of depression. Healthcare workers typically have poorer quality of life compared to other professions because they must deal with patients despite limited resources, multiple healthcare demands, low income, and many compounding factors [14]. Their quality of life is often negatively affected due to anxiety, depression, and lack social support from the people around them [10]. Also, one of the contributory factors to the high prevalence of depression among HCWs may have been due to the ongoing COVID-19 pandemic in Afghanistan. Research has shown that there has been a large impact on mental health among HCWs as a specific group during the pandemic particularly because of their higher exposure to the virus compared to other individuals [15].

The present study constitutes one of the very few attempts to evaluate the association between QOL and psychological distress among Afghan HCWs [16]. Quality of life is considered a factor beyond job satisfaction and is related to a person’s well-being [17]. Research has demonstrated that working conditions for healthcare professionals, mostly nurses and doctors, are subpar [18]. Many healthcare employees are mentally affected and the environment in which they operate is stressful by nature [17]. A study examining the Korean national database found that among HCW’s there were higher odds ratios for mood disorders (1.13, 95% confidence interval [CI] 1.11–1.15) and anxiety disorders (1.15, 95% CI 1.13–1.17) than employees in other industries [19]. Although the lifetime rates of depression among physicians appear to be similar to those of the general population, they are more prone to suicide [20].

As aforementioned, there was a high frequency of HCWs who reported depressive symptoms (73.6%). These results are in line with previous research that showed how distress, including anxiety, depression, alcoholism, and substance abuse, are more prevalent in Italy and Portugal among Italian and Portuguese HCWs and university personnel [17, 20]. Given the situation in the country, Afghan’s political circumstances have halted the availability of female literacy for years, resulting in fewer female HCWs [21]. Only two-fifths of the sample in the present study were female HCWs (39.5%). However, compared to other country-level states (22%), Herat’s female healthcare workers are almost twice in number [22]. Nine-tenths of HCWs reported that they earned below the equivalent of $100 (US) a month and a similar proportion of volunteers reported depression symptoms (90.3%). This suggests low income may be a factor for depression among HCWs that are additional to workload factors. The number of patients they attend to daily is another factor. Participants who attended to more than 50 patients a day reported a high level of depression compared to those who attended to lower numbers of patients. The findings are consistent with studies that show a high patient-HCW ratio as a contributing factor to depressive symptoms among HCWs [16]. While the aforementioned factors are related to the occurrence of depression, other factors such as relation to sedentary lifestyle, marital life, and family events are also reported to contribute to its occurrence in other studies [21].

Mental health problems among physicians constitute very important and under-estimated health factors because the well-being of HCWs appears to be an overlooked quality indicator of healthcare systems according to one review [23]. The WHO-QOL was used to assess QOL among HCWs. The finding that HCWs’ QOL was affected in the physical, psychological, social, and environmental domains corroborates previous research among Greek HCWs [24] reporting associations between depression and poor QOL [25]. Mental ill-health is associated with low QOL [26]. However, very few studies have addressed the influence of poor QOL on the HCWs’ work performance and further research is needed on this issue.

The present study also found a relationship between domains of quality of life (e.g., physical domain, psychological domain) and some of the socio-demographic related characteristics (e.g., the field of work, monthly income, job type) with depression symptoms. More than 90% of the participants with depressive symptoms stated that they earned below $100. McNeeley et al. reported that financial strain and lack of resources were associated with lower QOL [27]. This is an aspect that the governments should pay more attention to and assist employees with financial difficulties.

## Strengths and limitations

The strength of the present study was the utilization of standardized and validated instruments to assess the prevalence of depression symptoms and quality of life. However, there are a number of limitations. The study’s sample size was relatively low and may have been completed by a greater proportion of those who were unhappier with their job to start with. Moreover, the study only included hospitals in the urban area of Herat province so was not necessarily representative of hospitals in other areas or in other regions of Afghanistan. Also, due to the cross-sectional design of the study, the causality between depression symptoms and other variables could not be determined. Also, all the data were self-reported and are therefore subject to well-established methods biases. It should also be noted that there was no information available on number of medical doctors and/or other HCWs in Afghanistan because the country’s situation has changed a lot since August 2021 and hundreds of thousands of people left the country. Therefore, it is unknown what proportion of the eligible population participated in the present study (but was likely to be a very small percentage). Also, the present survey had only had two categories of HCW (i.e., doctors [including trainees] and non-doctors (nurses, midwives, etc.). Finally, over two-thirds of the collected data were from doctors (69.9%) rather than other types of HCW, therefore, this might have biased the findings to some extent.

## Conclusion

The prevalence of depression is high among healthcare workers in the Herat province of Afghanistan. One of the variables found to have a major impact on the prevalence of depression was their monthly income. Considering its impact on the quality of life and the overall quality of healthcare services, the government should implement regular screening for depression, psychological counseling services, and psychiatric treatment for vulnerable healthcare workers.

Considering the high prevalence of depression among women, the use of interventions including regular screening for depression symptoms, and educating women and girls on symptoms of depression for early self-diagnosis are essential. Also, due to the high prevalence of depression and anxiety, critical planning for providing psychiatric treatment for vulnerable is necessary.

## Data Availability

The datasets during and/or analyzed during the current study are available from the corresponding author on reasonable request.
